# Chabazite Synthesis and Its Exchange with Ti, Zn, Cu, Ag and Au for Efficient Photocatalytic Degradation of Methylene Blue Dye

**DOI:** 10.3390/ijms23031730

**Published:** 2022-02-02

**Authors:** José C. González-Crisostomo, Rigoberto López-Juárez, Rosario Isidro Yocupicio-Gaxiola, Eric Villanueva, Ethiel Zavala-Flores, Vitalii Petranovskii

**Affiliations:** 1 Centro de Nanociencias y Nanotecnología, Universidad Nacional Autonoma de México, Km 107 Carretera Tijuana-Ensenada, Ensenada 22800, Mexico; ryocu@cnyn.unam.mx (R.I.Y.-G.); vitalii@cnyn.unam.mx (V.P.); 2 Facultad de Ciencias Químicas e Ingeniería, Universidad Autónoma de Baja California, Calzada Tecnológico, Mesa de Otay, Tijuana 22390, Mexico; zavala.ethiel@uabc.edu.mx; 3 Instituto de Investigaciones en Materiales, Unidad Morelia, Universidad Nacional Autónoma de México, Antigua Carretera a Pátzcuaro, ExHacienda San José de la Huerta, Morelia 58190, Mexico; rlopez@iim.unam.mx; 4 Facultad de Ciencias de la Ingeniería y Tecnología, Unidad Valle de las Palmas, Universidad Autónoma de Baja California, Blvd. Universitario, Tijuana 2150018, Mexico; Eric.villanueva@uabc.edu.mx

**Keywords:** chabazite, hydrothermal method, silicates, methylene blue, photocatalysis

## Abstract

A chabazite-type zeolite was prepared by the hydrothermal method. Before ion exchange, the chabazite was activated with ammonium chloride (NH_4_Cl). The ion exchange process was carried out at a controlled temperature and constant stirring to obtain ion-exchanged chabazites of Ti^4+^ chabazite (TiCHA), Zn^2+^ chabazite (ZnCHA), Cu^2+^ chabazite (CuCHA), Ag^+^ chabazite (AgCHA) and Au^3+^ chabazite (AuCHA). Modified chabazite samples were characterized by X-ray diffraction (XRD), scanning electron microscope equipped with energy-dispersive spectroscopy (SEM-EDS), transmission electron microscopy (TEM), Fourier transform infrared (FTIR), N_2_ adsorption methods and UV–visible diffuse reflectance spectroscopy (DRS). XRD results revealed that the chabazite structure did not undergo any modification during the exchange treatments. The photocatalytic activity of chabazite samples was evaluated by the degradation of methylene blue (MB) in the presence of H_2_O_2_ under ultraviolet (UV) light illumination. The photodegradation results showed a higher degradation efficiency of modified chabazites, compared to the synthesized chabazite. CuCHA showed an efficiency of 98.92% in MB degradation, with a constant of k = 0.0266 min^−1^ following a first-order kinetic mechanism. Then, it was demonstrated that the modified chabazites could be used for the photodegradation of dyes.

## 1. Introduction

Currently, water scarcity in different areas of the world will be aggravated by 2050, due to the lack of rain, the lack of clean drinking water and the growing demand due to the increase in the world’s population [1,2]. On the other hand, water not only is essential for a sustainable, social and economic development but also for the environment and human beings’ survival. However, the scarcity of water is a growing threat for those topics and is worsened by the current pandemic situation of COVID-19 because water and sanitation are a main defense against the disease [3]. Water contamination is a global problem that results from pollutants such as oils, heavy metals, colorants (dyes and pigments), among others [4]. The colorants often come from industries such as textile, leather, paper, cosmetic, plastic and food; nevertheless, many of them are genotoxic, carcinogenic, poorly biodegradable with a high solubility in water [5]. Hence, it is important to get rid of colorants in water throughout different chemical and physical methods such as chemical oxidation, ozonation, membrane filtration, flotation and adsorption [6,7]. Dyes released to wastewater effluents from industries such as textiles, printing and cosmetics, among others, have become a serious environmental problem due to their high toxicity. Therefore, the removal of dyes from water and wastewater becomes a necessity, to develop environmentally good methods for the purification of contaminated water [8]. One of the dyes that cause harmful effects to human health and the environment is the buildup of methylene blue (MB) dye [9]. MB, C_16_H_18_ClN_3_S, a cationic colorant, is widely used in several technological fields, such as the textile industry, leather tanning, food processing, plastics, cosmetics, rubber, printing and in the manufacture of dyes. Dyes are not completely fixed in the products; they generate colored wastewater effluents, with a high organic content and low biodegradability, which leads to health problems because the dyes are toxic, recalcitrant, mutagenic and carcinogenic [10,11].

Zeolite chabazite is a tectosilicate mineral and is available in both natural and synthetic form. This structure has been assigned the three-letter code CHA by the International Zeolite Association (IZA) [12]. The chemical formula and crystalline structure of chabazite was first established by Dent and Smith [13,14,15] to be Ca_2_(Al_4_Si_8_O_24_)·12H_2_O. This zeolite is characterized by a low Si/Al ratio, which leads to a high ion exchange capacity. Structurally, chabazite belongs to the trigonal space group R3m [16]. The primary structural unit of all zeolites is Si or Al ions, tetrahedrally coordinated with four oxygen ions, each of which forms a bridge between neighboring tetrahedral atoms. These tetrahedra, connected by vertices, join together to form more complex secondary building blocks (SBUs), the combination of which ultimately results in a zeolite structure.

**Figure 1:**
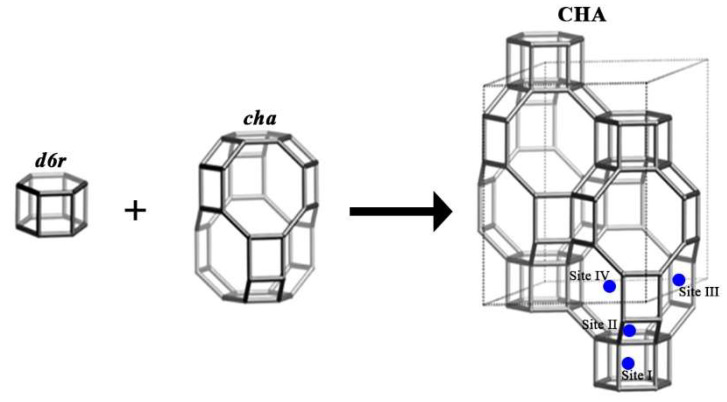
Composite building units and framework of chabazite viewed normal to [001] [12].

Chabazite consists of two main secondary building units, namely a hexagonal prism, formed by two parallel rings of six tetrahedra, called *d6r*, and a *cha* block, which contains rings of six and eight tetrahedra linked together (Figure 1). These two subunits form tertiary structures, which in turn combine to build the crystalline zeolitic structure of chabazite. *cha* blocks are joined to *d6r* blocks via six-member rings, these rings allow the connection to other *cha* blocks in the [001] direction, connecting to other *cha* blocks through an eight-member ring [17,18]. Chabazite is a zeolite with good cation exchange capacity. There are four different cationic sites in the chabazite structure: site I (hexagonal prism) in the center of a double six-ring prism, site II (supercage) on the axis of the six-ring prism triad, but shifted into the supercavity, site III in the supercavity and near the four-ring window, and site IV near the eight-ring opening [19].

The synthesis of chabazite is possible using a variety of methodologies, and not all mechanisms share the need for a template, seed or inducer in order to crystallize CHA-type zeolites. Basically, chabazite is synthesized from an inorganic gel, or by phase transformation of other zeolites under alkaline conditions; the resulting products are characterized by low Si/Al ratios [20]. When adding agents that can be used as a “template” to get crystal nuclei from the environment started and thus generate cavities and/or porosity “with characteristic dimensions”, organic cations with a specific geometry, such as N,N,N-1-trimethyladamantanammonium hydroxide (TMAdOH) [21] and tetraethylammonium hydroxide (TEA-OH) [22] are used as organic structure-directing agent (OSDA) for the chabazite formation. Interzeolite transformation is another effective approach to the selective synthesis of zeolites. The initial zeolite can first be decomposed into fragments containing structural elements common to many zeolites, and then the fragments can be rearranged and become the target zeolite.

The interzeolitic transformation of faujasite into chabazite can occur under hydrothermal conditions, due to the presence of SBU *d6r* common to both structures [23,24]. The process of ion exchange creates new bonds, causing the deformation of the initial structure of the zeolite [25]. To support of metallic nanoparticles using chabazite, the metallic cations located in the exchange positions can act as nucleation sites. The cages within the chabazite structure prevent particle cohesion, eliminating the tendency to form larger particles. The cations that remain on the outer surface of the chabazite can precipitate as metal or metal oxide nanoparticles. The treatment of chabazite with a solution of ammonium chloride (NH_4_Cl) before ion exchange leads to nanoparticles of controlled size [26,27].

In recent years, the application of photocatalysts on support materials has been studied, one of the main goals of which is to reduce electron–hole recombination as a determining factor in photocatalytic reactions. Zeolites are materials with good properties to act as a carrier [28,29]. Zeolites are an important group of heterogeneous industrial catalysts with large-scale applications in oil refining and petrochemistry and a growing potential for environmental catalysis [30]. For example, zeolites such as chabazite, zeolite Y, mordenite, faujasite and LTA have already been studied as catalyst carriers for the degradation of organic dyes [31,32,33]. In addition to the zeolite ZSM-12, which has been used in the photodegradation of MB with relatively low removal results [34], the synthesis of composite materials such as Zn-ZSM-5 [35], TiO_2_/Ni-ZSM-5 [36], g-C_3_N_4_-H-ZSM-5 [37] and clinoptilolite [38] improves the adsorption of MB to the zeolite surface, obtaining high photodegradation efficiencies.

In the present study, chabazite was synthesized as a support material by the hydrothermal method. Ion exchange was carried out with several transition metals (Ti, Cu, Zn, Ag and Au). A study was carried out on the photodegradation of MB in the presence of H_2_O_2_ under ultraviolet (UV) light irradiation, since illumination plays an important role in catalytic processes [39,40]. The synthesized materials were characterized in terms of their morphological, crystalline, optical and photocatalytic properties.

## 2. Results and Discussion

### 2.1. X-ray Analysis

**Figure 2:**
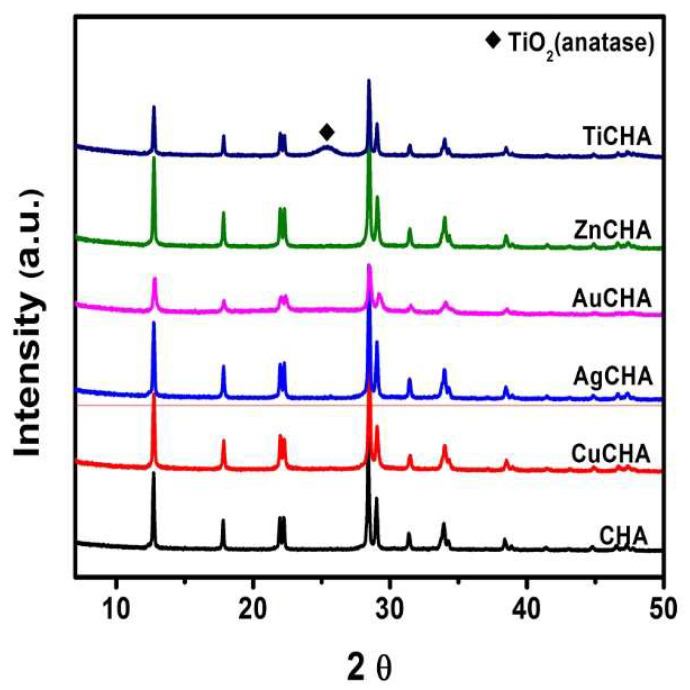
XRD patterns of the synthesized initial chabazite and ion-exchange-modified chabazites.

According to the X-ray diffraction results shown in Figure 2, the predominant crystalline phase found in the samples was a zeolite of the chabazite type. Clear signals on diffraction patterns at 2θ = 12.70°, 21.84° and 31.35° correspond to the main peaks of the chabazite-type structure, which have already been reported, indicating the presence of this structure [19,41]. From the experimental data presented, it can be seen that the incorporation of Ti, Zn, Cu, Ag, and Au does not lead to a loss of crystallinity, in contrast to other known observations in analogous cases during ion exchange for transition metals [42]. The chabazite synthesized in this work, with interplanar distances (in Å) corresponding to the planes (hkl) of 6.95 (−110), 4.97 (111), 4.04 (210), 3.99 (−211), 3.13 (300), 3.07 (−310), 2.85 (−131), 2.64 (−321), 2.34 (400), 2.31 (−410), 2.02 (−233) and 1.92 (−431) retains its crystallinity in the course of ion exchange. On the XRD diffraction patterns of all ion-exchanged powders, chabazite was found as the main phase. Additionally, a diffraction peak for TiO_2_ in the anatase phase, with a plane (1 1 1) at 25.3° was identified in the TiCHA sample [31]. It should also be noted that the intensities of the peaks of the TiCHA and AuCHA samples significantly decreased in comparison with the synthesized initial chabazite and other ion-exchange samples. The average grain size for all chabazite samples was obtained applying the Debye–Scherrer equation, which states that the grain size is inversely proportional to the average width of the maximum diffraction peak and the cosine of the maximum peak angle [43]. The average grain size for the chabazite samples was ~195 nm, except for the AuCHA sample for which it was ~81 nm.

### 2.2. SEM-EDS Analysis

**Figure 3:**
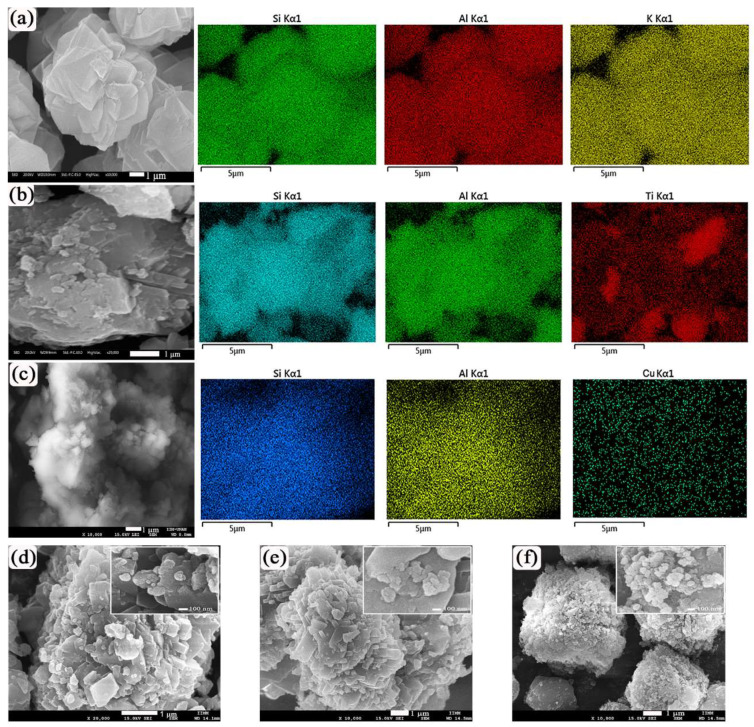
SEM images and EDS elemental mappings of (**a**) CHA (Si is shown in green, Al is shown in red and K is shown in yellow); (**b**) TiCHA (Si is shown in turquoise blue, Al is shown in green and Ti is shown in red); (**c**) CuCHA (Si is shown in blue, Al is shown in green and Cu is shown in yellow). (**d**–**f**) SEM images of ZnCHA, AgCHA and AuCHA, respectively.

Figure 3 shows SEM images of the initial synthesized chabazite samples and those modified by ion exchange. The SEM image of synthesized chabazite (Figure 3a) shows the resulting crystals of chabazite with a shape like a walnut, which is consistent with already published images [44]. The crystal has a size of about ~8 μm. An EDS mapping is also shown where it can be seen that there is a uniform distribution of Si (shown in green), Al (shown in red) and K (shown in yellow). TiCHA is shown in Figure 3b; particles with a size of ~5 µm are observed in it, and particles with a size of ~0.2–0.4 µm are observed on their surface. The distribution of titanium on the CHA surface (Ti is shown in red) is uniform. Figure 3c shows an SEM image for CuCHA and its respective EDS mapping for Si, Al and Cu. Particles with a size of ~5 μm are observed, and they contain small cubic particles with a size of ~0.6 µm. The distribution of copper is fairly uniform (shown in yellow). Figure 3d shows the morphology of the ZnCHA sample, where small particles with sizes ~40–90 nm can be observed, which represent the ZnO present on the CHA surface. The presence of silver nanoparticles on the CHA surface can be corroborated in Figure 3e. Particles with a size of ~5 nm are observed (image in the upper right corner). In Figure 3f one can see gold nanoparticles on the CHA surface; they are spherical and form aggregates with sizes of 40–120 nm.

The elemental composition of the synthesized and modified chabazite samples was determined by the EDS analysis. The results are shown in Table 1; the analysis showed that the cations Cu, Ag, Au, Zn and Ti were present in CHA after ion exchange. A decrease in the oxygen content was observed in the AgCHA and AuCHA samples, possibly due to the reduction of silver and gold on the CHA surface.

### 2.3. TEM Analysis

**Figure 4:**
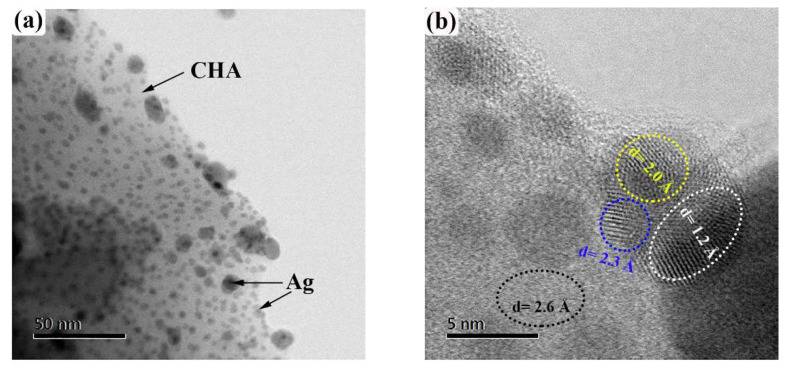

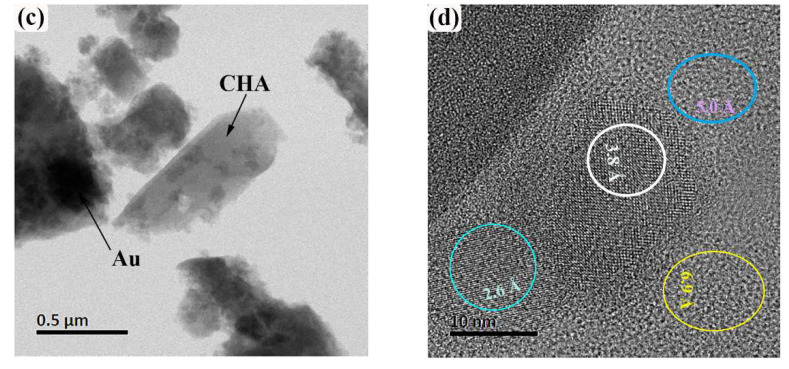
HRTEM micrographs of AgCHA (**b**) and AuCHA (**d**), and corresponding TEM images (**a**,**c**).

Figure 4 shows the morphology and structural parameters of AgCHA and AuCHA obtained using TEM. Ag and Au metal nanoparticles (dark spots) are observed on chabazite microcrystals. Figure 4a shows a micrograph of Ag nanoparticles supported on CHA. These particles are regularly distributed on the CHA surface as observed. The thermal calcination in an air atmosphere forced the exchanged silver ions to migrate to the surface, where they were reduced to neutral atoms and remained in the form of aggregated metallic nanoparticles [45]. The observed sizes of nanoparticle are in the range from 5 nm to 80 nm. At higher magnification in Figure 4a, it can be seen that the silver nanoparticles are crystalline. In Figure 4b, four crystalline planes can be clearly identified. The interplanar distances (ovals) for AgCHA are 2.3 Å, 2.0 Å, and 1.2 Å, which can be associated with planes (111), (200) and (311), respectively [46]. The found geometric and plane characteristics can be related to the fcc crystal structure of silver. EDS confirmed its presence, as shown in Table 1. The 2.6 Å interplanar spacing may be related to the (−321) CHA plane. Figure 4c shows a micrograph of Au nanoparticles supported on CHA, the sizes of Au nanoparticles are observed from 30 nm, consistent with the SEM image (Figure 3f). In Figure 4d, the interplanar distances for AuCHA are shown; they are 6.9 Å, 5.0 Å, 3.8 Å and 2.6 Å, associated with the planes of crystalline gold (−110), (111), (−211) and (−321), respectively.

### 2.4. FTIR Analysis

**Figure 5:**
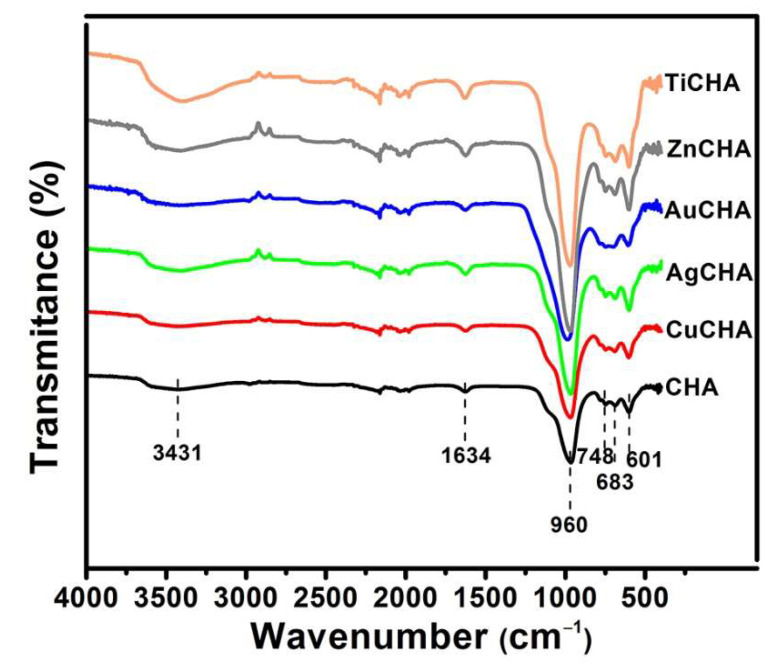
FTIR spectra of synthesized chabazite and modified chabazites.

The FTIR spectra of the original and modified chabazites were investigated in the range of 4000–400 cm^−1^ and are shown in Figure 5. The bands found show signals similar to those previously reported. The band at 3354 and 1634 cm^−1^ are attributed to stretching and bending vibrations of the O-H group, respectively [47]. The band at 960 cm^−1^ is associated with the asymmetric stretching vibration of Si-O-Si and Si-O-Al or with the stretching vibration of SiO_4_ in the chabazite structure. The bands at 683 and 748 cm^−1^ are attributed to the symmetric stretching vibration of Si-O-Si. The band at 601 cm^−1^ is related to the coupling vibration of the (SiO_4_)^4+^ and (AlO_4_)^5+^ tetrahedra [48,49,50]. The bands in the range of 800 to 500 cm^−1^, in addition to being related to the Si-O and Al-O tetrahedral units, are related to the sorption of metal cations [25]. In TiCHA, there was a slight shift of the peak at 960 cm^−1^ from the CHA at 969 cm^−1^, attributable to the Si-O-Ti bond. A certain amount of Ti could be incorporated into the *cha* structure during exchange treatment [51].

### 2.5. Specific Surface Area

The surface area of the synthesized and modified chabazite is shown in Table 2, along with the pore volume, average pore diameter and pore volume using the functional density theory (DFT) method. The DFT method estimates pore volume assuming that the layers accumulate in the pore wall until the pore is completely full. It can be expressed as a function of the density of the adsorbent species, at a given pressure [52,53]. As shown in Table 2, CHA gives a cumulative pore volume of 0.0004 cm^3^/g, a very small value compared to 0.0020 and 0.0061 cm^3^/g in AuCHA and TiCHA, respectively. This pore volume corresponds to a very low BET surface area of 1.08 m^2^/g. Although a BET surface area of 1 m^2^/g has already been reported for zeolite A of micrometric particle size by Torad, N.L. et al. [54]. Apparently, the opening of the *d6r* window is blocked by K^+^ ions to the point that the N_2_ molecules cannot penetrate the micropores. N_2_ molecules are excluded from CHA micropores, leading to a significantly lower N_2_ absorption. This explains the low surface area of CHA samples [55]. Furthermore, the dehydration of chabazite causes some distortion of the zeolitic structure of chabazite. Due to the molecular dimension of N_2_ (kinetic diameter of 0.364 nm) [56]. It is evident that the *d6r* unit is inaccessible to N_2_ even under dehydrated conditions, leaving only the eight-ring windows accessible. CHA has a considerably restricted pore size of 0.36 nm from the eight-ring window to the ellipsoidal cavity, the ionic radius of the cations being: K^+^ 0.133 nm, Cu^2+^ 0.072 nm, Ag^+^ 0.126 nm, Au^3+^ 0.085 nm, Zn^2+^ 0.074 nm and Ti^4+^ 0.068 nm [57]. Note the large size of the K^+^ cations blocking the micropores in the eight-ring window. Therefore, the N_2_ molecules cannot penetrate the CHA micropores, having a low N_2_ adsorption [55].

### 2.6. Visible Diffuse Reflectance Spectroscopy

**Figure 6:**
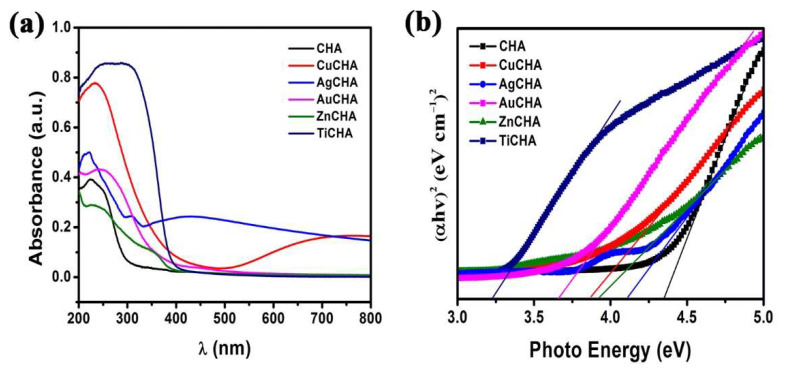
(**a**) UV–vis DRS and (**b**) band gap energies of synthesized chabazite and modified chabazites.

The diffuse reflectance absorption spectra of synthesized and modified chabazites are shown in Figure 6a. A UV–vis spectroscopy analysis was used to determine the current status of Ti, Zn, Cu, Ag and Au species. For the CuCHA sample the band at approximately 234 nm is attributed to the O → Cu^2+^ charge transfer [58]. The band at ~280–330 nm is attributed to the conversion of exchanged copper ions into oligonuclear [Cu-O-Cu]_n_ species, and the band at 330–420 nm to crystalline CuO_x_. The band at ~780 nm is attributed to the Cu^2+^ d-d (3 d^9^) transition [59,60,61]. In AgCHA, an absorption peak at ~430 nm is observed, indicating the presence of Ag nanoparticles [62]; the presence of silver cations (Ag^+^) is attributed to the observed peak at ~222 nm [63]. ZnCHA represents an absorption band at 330–390 nm, evidence of the presence of ZnO [64]. TiCHA had the highest absorption in the UV region compared to other modified chabazites. In the case of TiCHA, two absorption bands can be observed below and above 300 nm, called charge transfer bands. The absorption bands of Ti^4+^ ion (3d°) are observed at ~250 nm and 290 nm [65]. The charge transfer for TiO_4_ units is in the range of 200–260 nm; for the TiO_2_ lattice in the anatase phase, the charge transfer in TiO_6_ is greater than 300 nm [66]. The band gap E_g_ of synthesized chabazite and modified chabazites was calculated with the following known Equation (1):

(1) $$\alpha =\frac{A{\left(\mathrm{hv}-E_{g}\right)}^{1/2}}{\mathrm{hv}}$$

where α, E_g_ and A are the absorption coefficient, the band gap and the material parameter, respectively. By extrapolating the linear region of the plots (αhv)^2^ vs. hv (Figure 6b), the band gap of synthesized chabazite and modified chabazites was found. The results of the calculation of the band gap are shown in Figure 6b and Table 3. It can be seen that the band gap energy of the modified chabazites decreases with respect to the initial synthesized chabazite (E_g_ = 4.35 eV). This indicates that metal ions affect the band gap energy of chabazite. For TiCHA, the sample that presented the lowest value of band gap energy, it was 3.22 eV.

### 2.7. Photocatalytic Activity

**Figure 7:**
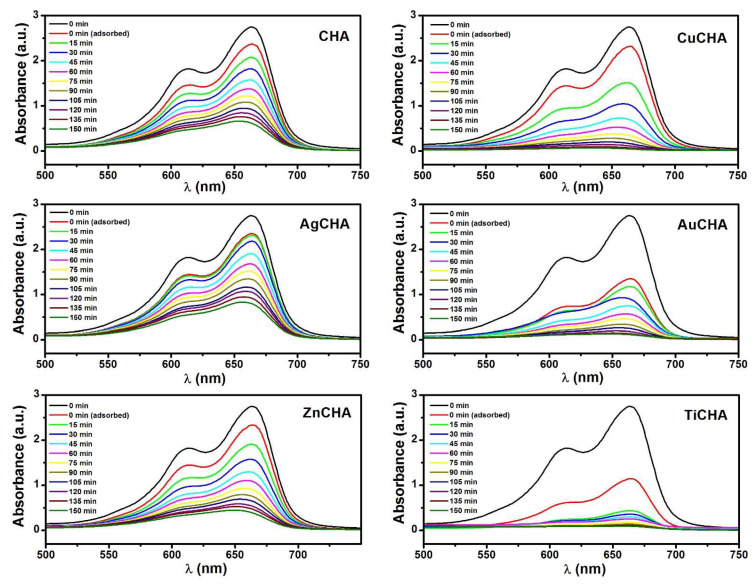
The change in absorption of methylene blue solution with time in the presence of H_2_O_2_ and synthesized chabazite and modified chabazites under UV irradiation.

The photocatalytic degradation activities of CHA, CuCHA, AgCHA, AuCHA, ZnCHA and TiCHA were evaluated in the presence of UV light and H_2_O_2_. The UV–vis absorption spectra of MB during degradation by catalytic samples at different irradiation times are shown in Figure 7. These optical absorption spectra correspond to aliquots of MB solutions taken at different times from 0 to 150 min. At 0 min, this corresponds to the absorption spectrum of the MB solution at the beginning of the experiment. The MB dye exhibits an absorption maximum at ~664 nm in the visible region [67]. It is observed that with an increase in the time of irradiation with UV light, the peak of maximum absorption at ~664 nm gradually decreases, and a more pronounced change is observed for CuCHA, AuCHA and TiCHA. The change in the color of the MB solution in the AuCHA and TiCHA samples during 30 min of darkness can be explained by the adsorption of MB molecules on the exposed open surface of the chabazite microcrystals [31]. Furthermore, in all photodegradation experiments, it can be observed that absorption bands change slightly at lower wavelengths over time. This blue shift in the wavelength of MB absorption bands can be attributed to the *N*-demethylation of MB molecules during photodegradation under the influence of UV light irradiation [68].

Based on the results obtained, a mechanism was proposed to explain the photocatalytic activity for synthesized and modified chabazites. For that, it was necessary to know the potentials of the valence band maximum (VBM) and conduction band minimum (CBM) of the synthesized and modified chabazites and they were estimated using the following equations:

(2) $$E_{\mathrm{CB}}=X-E_{C}-\frac{1}{2}E_{g}$$

(3) $$E_{\mathrm{VB}}=E_{\mathrm{CB}}+E_{g}$$

where E_VB_ and E_CB_ are the valence band and conduction band edge potentials, respectively. χ is the absolute electronegativity calculated from the geometric mean of the absolute electronegativity of the constituent atoms of the semiconductor material. E_C_ is the free electron energy on the hydrogen scale (4.5 eV) and E_g_ is the optical band gap of the material [69,70]. The VBM and CBM potentials versus normal hydrogen electrode (NHE) of the samples are listed in Table 3.

Chabazite acts as an absorber for MB molecules on its surface, thereby facilitating degradation by electrons promoted from the conduction (CB) to the valence band (VB). Electrons are generated in the conduction band (e_CB_^−^) and holes of electrons in the valence band (h_VB_^+^). The e_CB_^−^ located on the surface can react with O_2_ (redox potential: −0.33 eV vs. NHE) to produce the superoxide anion (O_2_·^-^), while h_VB_^+^ can react with H_2_O or OH^-^ bound on the surface to generate radical OH species (redox potential: 2.8 eV vs. NHE). O_2_·^-^ can react with H^+^ to produce H_2_O_2_ (redox potential: +0.682 eV vs. NHE), generate OH·, and these species can react with the MB dye to decompose it [71,72,73].

From the calculated potentials of VBM and CBM listed in Table 3, it can be affirmed that the photogenerated electrons on the CB of CHA, AgCHA, AuCHA and ZnCHA react with the absorbed O_2_ to form O_2_·^-^ radicals, because the CB of these samples is equal or more negative than the potential of O_2_/O_2_·^-^. On the other hand, the CB edge potential of CuCHA and TiCHA is more positive than the standard redox potential of O_2_/O_2_·^-^, therefore, the CB electrons of CuCHA and TiCHA cannot reduce O_2_ to O_2_·^-^. However, the electrons in the CB of CuCHA and TiCHA can be transferred to the adsorbed oxygen molecules to produce H_2_O_2_, because the CB levels of CuCHA and TiCHA are more negative than the redox potential of O_2_·^-^/H_2_O_2_. Subsequently, the H_2_O_2_ molecules produced react with the electrons to produce active OH· radicals, doing it in the same way as the samples CHA, AgCHA, AuCHA and ZnCHA. This suggests that MB photodegradation could be carried out by OH· radicals. General photocatalytic reactions caused by exposure to UV light irradiation to degrade the MB dye can be summarized as follows [74]:

(4) $$M\left(K,\mathrm{Cu},\mathrm{Ag},\;\mathrm{Au},\;\mathrm{Zn}\;\mathrm{or}\;\mathrm{Ti}\right)+\mathrm{hv}\to M(e_{\mathrm{CB}}^{-}+h_{\mathrm{VB}}^{+})$$

(5) $$H_{2}O+h_{\mathrm{VB}}^{+}\to \mathrm{OH}\cdot +H^{+}$$

(6) $$O_{2}+e_{\mathrm{CB}}^{-}\to O_{2}^{\cdot -}$$

(7) $$H^{+}+O_{2}^{\cdot -}\to H_{2}O_{2}$$

(8) $$H_{2}O_{2}+\mathrm{hv}\to 2\mathrm{OH}\cdot$$

(9) ·OH+MB→N−de−methylated intermediates→products

**Figure 8:**
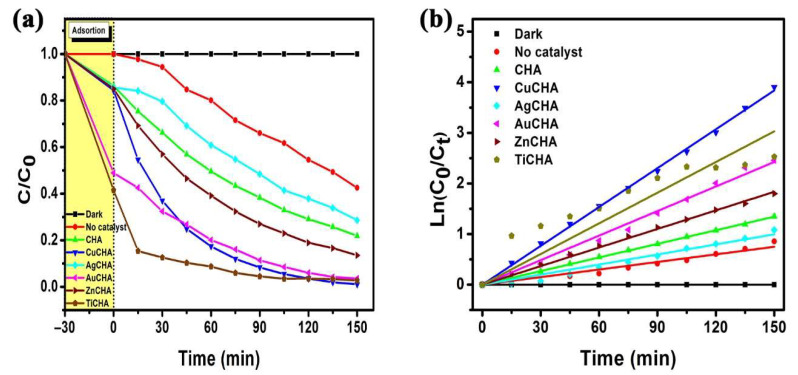
The photocatalytic degradation: (**a**) the degree of degradation of the methylene blue dye in each time interval; (**b**) a graph of the kinetics of the degradation of the methylene blue, plotted for the first linear order as ln(C_0_/C_t_) vs. irradiation time (t) in the presence of H_2_O_2_ and synthesized chabazite or modified chabazites under UV irradiation.

Figure 8a shows the change in the relative MB concentrations as a function of the time of exposure to UV light. The degradation rate of the MB dye is much lower in the AgCHA sample, which may be due to the low dispersion of Ag nanoparticles on the chabazite surface [75]; or, because MB is a cationic dye, it degrades little, due to the presence of Ag (Ag^+^) ions on the surface causing electrostatic repulsion between MB and AgCHA microcrystals, inhibiting the photodegradation process [76]. On the other hand, the photoreduction of Ag^+^ could be taking place, causing the elimination of e_CB_^−^, and in turn h_VB_^+^ would oxidize the silver nanoparticles, becoming a dominant process in the presence of oxygen, inhibiting the photodegradation of MB, as shown in Equations (10) and (11) [77].

(10) $${\mathrm{Ag}}^{+}+e_{\mathrm{CB}}{}^{-}\to {\mathrm{Ag}}^{0}$$

(11) $${\mathrm{Ag}}^{0}+h_{\mathrm{VB}}{}^{+}\to {\mathrm{Ag}}^{+}$$

Crystallinity is of great importance for catalytic performance, and when Ag cations are found, this leads to a considerable loss of catalytic activity [78]. The CuCHA sample showed the best degradation result, the degradation efficiency was 98.92%.

The results of MB degradation efficiency and first-order kinetic rate constants after 150 min of UV light irradiation in the presence of H_2_O_2_ are listed in Table 3 and shown in Figure 8b. The CuCHA sample showed the best result for photocatalytic activity, determined by the presence of Cu^+2^ ions, as seen from the DRS spectrum (Figure 6), which act as catalytically active centers. Furthermore, recent spectroscopic studies suggest that catalytically active di-copper species with oxygen bridges (dimers) may exist in CuCHA materials [79,80]. This may be since initially copper is partially hydrated, and at the time of heat treatment at 450 °C, water molecules are removed from CHA and Cu^+2^ ions move towards the six-member ring of CHA due to the electronegativity of oxygen atoms [81].

## 3. Materials and Methods

### 3.1. Materials and Reagents

Sodium hydroxide (NaOH, 98%), sodium aluminate (NaAlO_2_, 53 wt % of Al_2_O_3_, 42.5 wt % of Na_2_O), sodium silicate solution (25 wt % of SiO_2_ and 10.6 wt % of Na_2_O), potassium hydroxide (KOH, 90%), ammonium chloride (NH_4_Cl, 99.5%), copper nitrate trihydrate (Cu(NO_3_)_2_·3H_2_O, 99%), silver nitrate (AgNO_3_, 99%), Au solution (47 mg/mL), zinc nitrate hexahydrate (Zn(NO_3_)_2_·6H_2_O, 98%), titanium isopropoxide (C_12_H_28_O_4_Ti, 97%) were supplied by Sigma-Aldrich, St. Louis, MO, USA. Methylene blue and hydrogen peroxide (H_2_O_2_, 50 wt %) were supplied by Jalmek Científica, San Nicolas de los Garza, N.L., Mexico.

### 3.2. Synthesis of Chabazite

For the synthesis of chabazite, an interzeolitic transformation was chosen. First, a Y-type zeolite was synthesized by the hydrothermal method, described by Ginter et al. [82]. The prepared seed gel (19.95 g of water, 4.07 g of NaOH, 2.09 g of NaAlO_2_ and 22.72 g of sodium silicate solution containing 25% by weight of SiO_2_ and 10.6% by weight of Na_2_O) was stirred for 10 min and incubated at room temperature for 24 h. Then, a gel was prepared for the synthesis of the starting material (130.97 g of water, 0.14 g of NaOH, 13.09 g of NaAlO_2_ and 142.43 g of sodium silicate solution containing 25% by weight of SiO_2_ and 10.6% by weight of Na_2_O), which was stirred vigorously for 20 min. To the gel thus prepared, 16.5 g of the seed gel was added and the resulting mixture was stirred for an additional 20 min. Finally, the crystallization process was carried out in a polypropylene bottle, for 24 h at 100 °C. The resulting product (Y-type zeolite) was recovered by filtration, washed with distilled water and dried at 110 °C.

Chabazite was synthesized using the procedure described by Bourgogne et al. [83]. For this, 198.2 mL of water, 26.8 mL of a 45% by weight KOH solution were added to 25 g of Y-zeolite, and the resulting mixture was stirred for 30 s. Crystallization was carried out in a polypropylene pot, at 95 °C for 96 h. The product was recovered by filtration, washed with distilled water, and dried at room temperature.

### 3.3. Ion Exchange Process in the Synthesized Chabazite

For the ion exchange process, chabazite was preconditioned with multiple processes (six) of ion exchange NH_4_^+^ .In each exchange process, chabazite powder was refluxed in a 0.5 M NH_4_Cl solution for 1 hour (1 g chabazite/10 mL of solution). At the end of each step, the mixture was filtered and washed. At the end of the entire process, the resulting chabazite was filtered, thoroughly washed, and dried at room temperature.

The NH_4_-chabazite prepared in this way was used in the processes of exchange for copper, silver, gold, zinc and titanium. In each exchange, 2 g of conditioned chabazite and 40 mL of 0.01 M solutions of Cu(NO_3_)_2_·3H_2_O, AgNO_3_, Zn(NO_3_)_2_·6H_2_O and titanium isopropoxide were used. To prepare a gold-containing sample, 4.17 mL of a solution containing 47 mg/mL of Au was used. The exchange was carried out at 70 °C with constant stirring for 24 h. After the exchange, the products were filtered, and washed thoroughly with water, and dried at 80 °C overnight. Then, the samples were placed in a furnace (Thermolyne-46100) and heat-treated at 450 °C for 4 h in an air atmosphere, with a linear heating and cooling ramp of 5 °C min^−1^.

Through text and figures, the end products are labeled as CuCHA, AgCHA, AuCHA, ZnCHA and TiCHA.

### 3.4. Characterization Methods

The crystalline phases of the obtained samples were characterized by X-ray diffraction. A Philips X-Pert MPD diffractometer was used, applying a scan step of 0.020°, a step time of 1.2 s per data point, with a Cu tube to generate K_α_ radiation (λ = 1.54056 Å), in the 2-theta range from 5° to 50°. The morphology and structure of the synthesized samples were analyzed using a JEOL JSM-IT300 scanning electron microscope and a JEOL JEM-ARM200F transmission electron microscope. To assess the presence of functional groups in the original and modified chabazites, Fourier transform IR spectra were recorded in the wavenumber range from 4000 to 400 cm^−1^ using an FTIR OMNIC 9 infrared spectrometer from Thermo Fisher Scientific Inc. The specific surface area, total pore area and total pore volume of the synthesized nanoparticles were determined by measurements of nitrogen adsorption–desorption isotherms at −196 °C taking P/P_0_ from 0 to 0.99, using a Micromeritics Tristar II apparatus on degassed samples at 300 °C under vacuum for 3 h. The specific surface area was calculated by the Brunauer–Emmett–Teller (BET) method between 0.1 and 0.3 relative pressure. Pore volume was determined by the functional density theory (DFT) method. Measurements of the UV–vis diffuse reflectance spectroscopy (DRS) of the obtained materials were performed in the wavelength range from 200 to 800 nm using a Cary 5000 spectrometer. The UV–vis measurements of the products of photocatalytic decomposition of MB were recorded in the wavelength range from 500 to 750 nm using a spectrometer Hach DR 5000.

### 3.5. Photocatalytic Degradation of Methylene Blue

The photocatalytic properties of the synthesized materials were evaluated in the decomposition reaction of the methylene blue (MB) dye, which was carried out in a Pyrex vessel-type reactor, under UV illumination using a UVP UVGL-58 UV lamp, 254/365 nm, 6W. The lamp was used at 254 nm having an irradiance of 1356 μW cm^−2^. Solutions of MB with a concentration of 1 × 10^−4^ mol L^−1^ were used. To 80 mL of the solution, 80 mg of each of the test materials and 1.0 mL of H_2_O_2_ were added. Prior to irradiation with UV light, the suspension was continuously stirred for 30 min in the dark to achieve an adsorption–desorption balance between the dye and the photocatalyst. Then, the suspensions were irradiated with UV light with constant stirring. The samples were periodically removed every 15 min and centrifuged at 3000 rpm to separate the photocatalyst. The kinetic model of photocatalytic reduction of MB is described by the Langmuir–Hinshelwood kinetic model (L–H), which can be expressed with the following first-order equation [31,84]:

(12) $$\ln\left(\frac{C_{0}}{C_{t}}\right)=k_{\mathrm{app}}t$$

where C_0_ is the initial concentration of MB, C_t_ is the concentration of MB at any time, *t* is the illumination time (min) and k is the apparent velocity constant (L/min). The degradation rate was calculated using the following equation:

(13) $$Dye\;degradation\;efficiency=\left(1-\frac{C}{C_{0}}\right)\times 100$$

where C and C_0_ are the final and initial concentration of the MB dye in the solution.

## 4. Conclusions

In this work, undoped and modified chabazites were synthesized by the hydrothermal method. The ion exchange process was carried out at a controlled temperature and constant stirring, producing ion-exchanged chabazites TiCHA, ZnCHA, CuCHA, AgCHA and AuCHA, which were then heat-treated at 450 °C. Changes in the structure of the synthesized and modified chabazites were analyzed with XRD, SEM-EDS, TEM, FTIR and UV–vis DRS. XRD analysis showed the absence of structural changes of the chabazite during the ion exchange process, chabazite being the main phase, with the exception of TiCHA, where the TiO_2_ impurity in the anatase phase was identified. BET surface area results confirmed that N_2_ adsorption at −196 °C does not adequately show the textural properties of CHA due to pore blockage. The photocatalytic activity of the original synthesized and modified chabazites was estimated by the degradation of methylene blue, a cationic dye, which is difficult to biodegrade. The results of MB photodegradation results were quite good: the efficiency and degradation rate constant varied from 71.37% and k = 0.0066 min^−1^ to 98.92% and k = 0.0266 min^−1^ for AgCHA and CuCHA, respectively. Therefore, the present investigation provides a solution to treating water contaminated by dyes.

## Figures and Tables

**Table 1:** Average elemental composition of synthesized and modified chabazite samples.

Atomic, %	CHA	CuCHA	AgCHA	AuCHA	ZnCHA	TiCHA
O	68.89	68.00	48.46	55.62	60.61	70.51
Si	15.40	17.19	26.60	23.54	19.41	13.70
Al	8.52	8.67	14.76	18.15	16.69	7.64
K	7.20	5.95	9.17	2.64	2.59	5.69
Cu	--	0.18	--	--	--	--
Ag	--	--	1.00	--	--	--
Au	--	--	--	0.04	--	--
Zn	--	--	--	--	0.69	--
Ti	--	--	--	--	--	2.47

**Table 2:** Pore and surface area properties for synthesized chabazite and modified chabazites.

Samples	Surface Area BET (m^2^/g)	Cumulative Surface Area of Pores (m^2^/g)		Total Pore Volume (cm^3^/g)	Average Pore Diameter ^a^(nm)	Pores Volume DFT (cm^3^/g)
		Adsorption	Desorption			
CHA	1.08	0.45	0.26	0.0004	1.32	0.0004
CuCHA	2.70	1.92	1.36	0.0025	3.70	0.0009
AgCHA	1.12	0.10	0.34	0.0003	1.07	0.0003
AuCHA	20.39	21.26	19.34	0.0054	1.07	0.0020
ZnCHA	2.81	2.33	1.50	0.0018	2.52	0.0005
TiCHA	52.75	56.96	61.52	0.0528	4.00	0.0061

^a^ Obtained for 4 V/A by BET, where V is the total pore volume and A is the surface area.

**Table 3:** Degradation efficiency (%) and pseudo first-order rate constant (κ) for the photocatalytic degradation of methylene blue using synthesized chabazite or modified chabazites.

Samples	Band Gap (eV)	E_VB_ (eV)	E_CB_ (eV)	Degradation Efficiency (%)	κ (min^−1^)	R^2^
CHA	4.35	4.01	−0.33	78.11	0.0090	0.9998
CuCHA	3.86	3.76	−0.10	98.92	0.0266	0.9965
AgCHA	4.10	2.83	−1.27	71.37	0.0066	0.9937
AuCHA	3.65	3.11	−0.54	96.39	0.0162	0.9961
ZnCHA	3.92	3.51	−0.41	86.46	0.0122	0.9993
TiCHA	3.22	3.53	+0.31	97.27	0.0202	0.9542

## Data Availability

The raw/processed data required to reproduce these findings cannot be shared at this time as they also form part of an ongoing study.
